# Evaluation of the Effectiveness of a School-Based Smoking Prevention Program Among Young Adolescents in Central Greece: An Analytical, Non-Randomized Interventional Study

**DOI:** 10.3390/ijerph23020270

**Published:** 2026-02-22

**Authors:** Sofia Maria Panagiotidou, Maria Tziastoudi, Marios Politis, Chrissi Hatzoglou, Ioannis Stefanidis, Panagiotis Behrakis, Christos Hadjichristodoulou, Georgios Rachiotis

**Affiliations:** 1 Department of Hygiene and Epidemiology, Faculty of Medicine, School of Health Sciences, University of Thessaly, 41222 Larissa, Greece; 2 Department of Nephrology, Faculty of Medicine, School of Health Sciences, University of Thessaly, 41110 Larissa, Greece; 3 Department of Physiology, Faculty of Medicine, School of Health Sciences, University of Thessaly, 41110 Larissa, Greece; 4 SmokeFreeGreece, Hellenic Society Against Cancer, 11521 Athens, Greece

**Keywords:** smoking prevention, school-based intervention, adolescents

## Abstract

**Highlights:**

**Public health relevance—How does this work relate to a public health issue?**

- Smoking initiation occurs early in adolescence, contributing to long-term tobacco-related morbidity and mortality.
- Yet, relevant research focused solely on elementary school students is limited.

**Public health significance—Why is this work of significance to public health?**

- The findings of this study contribute to the international literature on early smoking prevention. The findings demonstrate that preventive interventions are effective even before regular smoking behaviors are established.
- By evaluating school-based intervention among elementary school students using a longitudinal, interventional design, this study adds evidence from a developmental stage that remains underrepresented in global tobacco prevention research

**Public health implications—What are the key implications or messages for practitioners, policy makers and/or researchers in public health?**

- Integrating structured smoking prevention programs into primary school curricula can strengthen early tobacco control strategies.
- The characteristics of our intervention (brief and delivered within routine school settings) enhance its potential applicability to other countries, particularly those with high adult smoking prevalence or national settings with limited resources.

**Abstract:**

Background: Smoking remains a major global public health challenge. As smoking often begins in early adolescence, early preventive programs are essential, yet research focusing exclusively on elementary school students is limited. This study measured smoking prevalence, smoking-related knowledge and attitudes, and the impact of a school-based intervention on these outcomes among 12–13-year-old students in Larissa, Greece. Methods: A total of 769 students participated (response rate: 75%). Knowledge, attitudes, and smoking prevalence were assessed at baseline. The intervention group (*n* = 316) was exposed to audiovisual and printed materials, and both groups were followed up at three- and twelve months post-intervention. Multivariable linear mixed-effects models and generalized estimating equations were used to evaluate intervention effects. Results: Baseline characteristics were balanced between groups. A statistically significant stage × group interaction was observed, indicating improvements in smoking-related knowledge and attitudes (*p* < 0.001) and a reduced likelihood of smoking initiation (*p* = 0.011) in the intervention group. Conclusions: This school-based intervention demonstrated significant improvements in knowledge and attitudes toward smoking and reduced the likelihood of smoking initiation. These findings support integrating early prevention programs into school curricula as a potentially effective approach to improving smoking-related outcomes.

## 1. Introduction

Smoking, a global epidemic and a major public health threat, is responsible for the death of 7 million people each year [1,2]. Tobacco use is widespread globally but unevenly distributed, with 80% of the 1.3 billion tobacco users currently living in low- and middle-income countries [1]. Smoking is responsible for 90% of lung cancer cases, about 85–90% of all chronic obstructive pulmonary disease (COPD) cases and is a major risk factor for ischemic heart disease, stroke, asthma, and other conditions [3,4]. Early age of cigarette smoking initiation has been associated with greater smoking-associated long-term health risks and dependence [5]. Given this, adolescents and young adults are particularly vulnerable to the risks of smoking due to the longer expected duration of tobacco use [6].

Despite some conflicting evidence, school-based smoking prevention programs have, in many cases, demonstrated effectiveness in achieving both short- and long-term outcomes [7,8,9,10,11,12]. More specifically, these programs have been reported to prevent adolescent cigarette use, reduce smoking prevalence, and lower the likelihood of smoking initiation among this group [9,10,11,13,14,15]. Evidence suggests that school-based programs can influence smoking-related attitudes, beliefs, and behaviors by promoting healthy lifestyle choices, increasing awareness of the dangers of tobacco use, and equipping students with the skills to resist social pressures to smoke [16].

Although school-based smoking prevention has been widely studied, most interventions internationally target adolescents in early or middle secondary education, often after experimentation with tobacco has already begun. In contrast, research focusing specifically on elementary school-aged students, prior to smoking initiation, remains comparatively limited. Early adolescence represents a critical developmental period during which attitudes, beliefs, and intentions related to health behaviors are formed. Interventions delivered at this stage may therefore have greater long-term preventive potential, yet relevant research focused solely on elementary school students is limited. Addressing this gap is of international relevance, as early prevention strategies are increasingly recognized as a crucial stage in smoking prevention.

Given the aforementioned evidence, the aim of the present study was to assess the prevalence of smoking, as well as participants’ knowledge and attitudes toward smoking, and to evaluate the effectiveness of a school-based intervention in reducing the likelihood of smoking initiation and improving knowledge and attitudes among the participants. Specifically, this study sought to address the following research questions:

1. What is the prevalence of smoking among participants before the intervention?
2. What are the levels of knowledge and attitudes toward smoking among participants before the intervention?
3. Is the school-based intervention effective in reducing the likelihood of smoking initiation and improving knowledge and attitudes toward smoking among participants?

## 2. Materials and Methods

### 2.1. Population—Inclusion Criteria

The present study was an analytical, non-randomized interventional study with control group, conducted among 6th-grade students in Larisa, Greece, during the 2017–2018 academic year. In that year, the total number of 6th-grade students in the city was 1667. Eligibility criteria for participation included enrolment in the 6th grade during the 2017–2018 academic year and provision of parental consent.

### 2.2. Sampling 

The sample size was calculated using the standard formula for comparing two independent proportions, based on the expected prevalence of smoking at the end of the study: 8% in the intervention group and 16% in the control group [17]. The prevalence estimate for the control group was based on data from the Global Youth Tobacco Survey (GYTS) 2013 conducted in Greece [18], findings that were supported by later national survey results reporting that 16% of students aged 13–15 years had used any tobacco product [19].

The calculation assumed a power of 80%, a 95% significance level, a response rate of 90%, a design effect of 1.5 to account for clustering, and an allocation ratio of 1:2 (intervention: control). A 1:2 allocation ratio was selected to enhance statistical efficiency while addressing practical constraints. A larger control arm improves the precision of baseline risk estimation, thereby increasing overall study power without the need to recruit additional participants. In parallel, resource limitations informed this choice, as delivery of the 2 h intervention program requires trained facilitators and dedicated materials.

The study employed a two-stage sampling design. In the first stage, schools were selected through probability sampling, and in the second stage, students were chosen using non-probability methods. For the school sample, stratified random sampling was applied to capture differences in geographical context (urban, semi-urban, and rural). Within each stratum, a unique random number was assigned to every school in the sampling frame, and schools were then sorted according to these random numbers. Participation was voluntary, and students were selected based on their availability during the researcher’s visits. Participation of the students in the study required written informed consent from their parents or legal guardians

### 2.3. Questionnaire

A questionnaire with closed-ended questions was developed based on a review of relevant literature and by appropriately adapting previously validated instruments to the specific context of this study [20,21]. The questionnaire comprised three thematic sections: (1) sociodemographic characteristics, (2) knowledge and attitudes toward smoking and (3) tobacco use (see Supplementary Materials).

### 2.4. Pilot Study

Initially, the process of organizing the research was evaluated through a pilot study, focusing on the method of distributing and completing the questionnaire. The purpose of this pilot study was to identify potential comprehension problems by observing respondents’ reactions and reviewing their answers. These issues could relate to the form and language of the questionnaire or to unclear or confusing points. The pilot study was conducted with a total of 20 students. Afterwards, the students were asked to provide feedback on the clarity of the questions and to report any difficulties encountered while answering. Cronbach’s alpha was calculated to assess the internal consistency of the knowledge–attitude scale and was found to be 0.73, indicating acceptable internal consistency.

### 2.5. Intervention

Participants in the intervention group received a two-hour educational session, with a baseline assessment of their attitudes and smoking habits conducted prior to the intervention. Follow-up assessments were carried out three months after the intervention and again one year later, resulting in a total of three measurement points: at baseline, three months post-intervention (in the sixth grade), and one-year post-intervention (in the first grade of middle school).

In the first phase, sixth-grade students from both intervention and control schools completed a baseline questionnaire assessing their knowledge, attitudes, and practices regarding smoking prior to the educational intervention. Immediately afterward, the intervention group received a two-hour educational session based on the HEART II program and the “I Learn the Truth—I Say No to Cigarettes” initiative of the Institute of Public Health of the American College of Greece, incorporating audiovisual and printed materials as well as age-appropriate questionnaires [22]. In the second phase, approximately three months later, the same students completed the questionnaire again to evaluate changes in their smoking-related knowledge, attitudes, and practices. During the third phase, about one year after the intervention, the students were followed up in Larissa’s middle schools to assess these outcomes, allowing comparison between the intervention and control groups over time.

### 2.6. Data Collection

After securing the necessary approvals, the researcher visited the school premises and distributed the questionnaires. Each questionnaire was accompanied by a letter providing the researcher’s details, the purpose of the study, and assurances of anonymity, confidentiality, and voluntary participation. The distribution and collection of the questionnaires took place in the presence of the researcher, both before and after the intervention. Completion of the questionnaire required no more than 30 min.

### 2.7. Independent Variables

As independent variables in the multivariable model, we included age, sex, and variables derived from the first section of the questionnaire regarding the smoking habits of participants’ family members and friends. Specifically, students were asked whether their mother, father, or any siblings smoke, as well as whether any of their friend’s smoke. The possible responses for each question were “Yes,” “No,” “I do not know,” or “I do not have/does not live with me.”

### 2.8. Dependent Variables

The 16 items in the second section, which assessed participants’ attitudes and knowledge about smoking, were combined in a logical manner (from negative to positive attitudes and from low to high knowledge) to create a continuous variable, “smoking knowledge–attitudes,” ranging from 0 to 80.

Although three distinct questions on smoking prevalence were included in the questionnaire (see Supplementary Materials), a measure of smoking in the past 30 days was used for the analysis, in accordance with the GYTS definition [23]. Smoking prevalence during the past 30 days was assessed with the question: “Have you smoked at all during the past month (even a single puff)?”.

### 2.9. Statistical Analysis

All statistical analyses were performed using SPSS version 29. A complete case analysis was conducted. Age is presented in years with the corresponding standard deviation (SD), while categorical variables are presented as frequencies with corresponding percentages. To evaluate baseline imbalances between the control and intervention groups, standardized differences for means and prevalence were calculated for all variables. Standardized difference values were interpreted using Cohen’s rule of thumb, with 0.2 indicating a small difference, 0.5 a medium difference, and 0.8 a large difference [24]. Baseline standardized differences were calculated according to the formulas described by Peter C. Austin [25].

The knowledge–attitudes scale was treated as a continuous variable and summarized as mean (SD), while smoking prevalence was summarized as a percentage. Intervention effects were assessed using a multivariable linear mixed-effects regression model for the knowledge–attitudes scale and a multivariable generalized estimating equations (GEE) model for smoking prevalence. For the knowledge–attitudes scale, stage, sex, and the stage × group interaction were included and adjusted for group, age, maternal smoking, paternal smoking, and sibling smoking. Given the low number of smoking initiation events, model complexity for smoking prevalence was restricted to reduce the risk of overfitting. A parsimonious two-level GEE model was therefore fitted, including group, stage, and their interaction. Additional covariate adjustment was limited to age and sex. Repeated measures and clustering were accounted for using two-level mixed-effects models with participants nested within schools for the knowledge–attitudes scale, and GEE with robust standard errors for smoking prevalence.

### 2.10. Ethical Issues

Participation of the students in the study required written informed consent from their parents or legal guardians. The protocol of the study has been approved by the Ministry of Education, Religious Affairs and Sports (57683/12 April 2018), the Institute of Educational Policy (4073/12 April 2018), as well as the General Assembly of the Faculty of Medicine of the University of Thessaly (5262/11 October 2017).

## 3. Results

**Figure 1:**
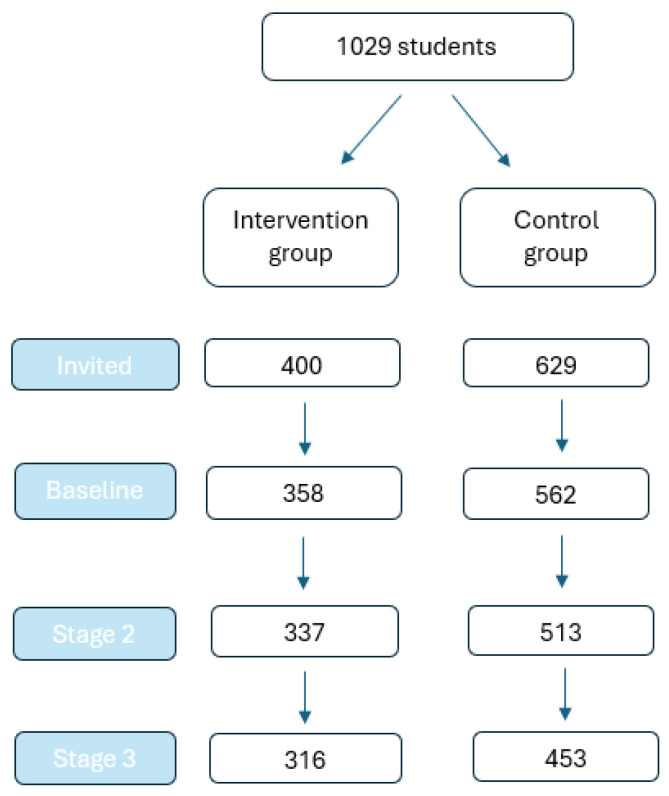
Flow of study participants from invitation through baseline and follow-up (Stages 1 and 2).

A total of 1029 students were invited to participate in the study, with 400 allocated to the intervention group and 629 to the control group (Figure 1). At baseline, 358 students in the intervention group and 562 in the control group were included. By Stage 2, 316 students in the intervention group and 453 in the control group completed the study and were available for analysis. Overall, 769 students participated, yielding a response rate of 75%. Between baseline and Stage 2, loss to follow-up occurred in 21% of participants in the intervention group and 28% in the control group.

Baseline characteristics of study participants are presented in Table 1. Overall, the groups were well balanced, with only minor differences observed for paternal and maternal smoking, which were slightly more prevalent in the intervention group.

Table 2 presents the scores of the smoking knowledge-attitude scale across the three stages of the intervention in both the control and intervention groups. The observed range of scores was between 33 and 77 out of 80. A slight improvement was observed in the intervention group, with scores increasing from 60.36 at baseline to 62.27 at stage 3, while no notable changes were observed in the control group.

According to the multivariable mixed-effects linear regression model, the interaction between stage and group was statistically significant (*p* < 0.001), indicating that changes over time in knowledge and attitudes toward smoking differed between the two groups (Table 3).

Table 4 presents the prevalence of smoking at the three stages of the study in both the intervention and non-intervention groups. Although participants in the control group started with a lower baseline prevalence compared to the intervention group (0.22% and 0.51%, respectively), by stage 3 the smoking prevalence was 2.68% in the control group and 1.81% in the intervention group.

According to the multivariable GEE model, the stage × group interaction was statistically significant (*p* = 0.011), indicating that changes in smoking prevalence over time differed between the two groups (Table 5).

## 4. Discussion

In this study, participants in both groups demonstrated good knowledge and positive attitudes toward smoking prior to the intervention. Baseline smoking prevalence was 0.51% in the intervention group and 0.22% in the control group. A statistically significant stage × group interaction was observed, indicating that the intervention was associated with improvements in knowledge–attitudes scores and lower smoking initiation among participants in the intervention group compared with the control group.

There are meta-analytic lines of evidence suggesting that smoking intervention programs significantly improved smoking behaviors and prevented smoking initiation among young adolescents with both short- and long-term effects taken into account [10,11]. The most effective interventions were school-based programs, particularly those delivered by trained teachers [11]. High-intensity programs, also resulted in a significant reduction in smoking use [11]. In addition, X:IT intervention a large prospective study in Denmark the X:IT intervention included also core systematic features of an antismoking campaign like smoke-free school grounds, a smoke-free curriculum, and parental involvement, which collectively contributed to the control and decrease of smoking use among adolescents [26]. Mpousiou et al. in a school-based interventional study in Athens, Greece used ancient Greek myths to convey anti-smoking messages to middle-school students [27]. Among 351 participants, those in the intervention group showed better knowledge, stronger anti-smoking attitudes, and less intention to smoke after 3 months, in comparison to the control group [27].

Although smoking initiation often occurs during adolescence, our findings show that preventive education can be effective even before most students begin experimenting with tobacco. Implementing school-based interventions at the primary level may therefore serve as a key strategy for the primary prevention of smoking. This is of importance since Greece continues to report one of the highest adolescent and adult smoking rates in Europe [28,29]. Alternatively, integrating structured, evidence-based prevention modules into national curricula could enhance the primary prevention of tobacco use at an early age. Early prevention also aligns with Greece’s National Action Plan for Public Health [30]. Beyond the Greek context, the results of the present study may have implications for early smoking prevention policies in countries with similarly high adult smoking prevalence, comparable national-level resources, and similar cultural backgrounds. In this light, the characteristics of our intervention—brief and delivered within routine school settings—enhance its potential applicability to similar settings.

The observed improvements in students’ knowledge and attitudes toward smoking and prevalence of smoking may be interpreted through the optic of Bandura’s Social Cognitive Theory [31], and the Theory of Planned Behavior [32] which focus on three independent determinants of intention (attitudes towards the behavior under study; subjective form which is reflects the perceived social pressure to accept or reject the behavior; and also the degree of perceived behavioral control (self-efficacy). The educational intervention—e.g., by providing audiovisual material, guided discussion, and peer interaction—may have created opportunities for observational learning, where students could model anti-smoking behaviors and recognize positive social norms associated with tobacco refusal. Through exposure to persuasive messages and the use of relatable examples, the program likely enhanced students’ self-efficacy—their belief in their ability to resist social pressure to use tobacco and nicotine products. The above-mentioned mechanisms collectively help explain why knowledge and attitudes substantially improved, and why the intervention was able to effectively control the prevalence of smoking over time. Finally, it is important to place the observed changes in smoking-related knowledge and attitudes into a clinically meaningful context. The intervention arm showed an average increase of approximately 2 points at both 3 and 12 months post-intervention. While this magnitude of change may appear small to modest in absolute terms, it may still have practical significance in a pre-teen population. Even modest improvements in knowledge and anti-smoking attitudes can strengthen resistance to smoking initiation pressures and contribute to long-term behavioral protection, particularly in low-prevalence settings. Although the effects of a brief 2 h intervention might be expected to attenuate over time, the persistence of a 2-point improvement at both follow-up points suggests enhanced resilience to future smoking risk in the intervention group compared with controls.

### Strengths and Limitations

A key strength of our study is that the statistical analysis accounted for differential effects over time through the interaction between stage and participant group. Furthermore, our sample consisted of elementary school students, an underrepresented population in global tobacco prevention research; therefore, beyond its relevance to the Greek context, the findings contribute to the international literature on early smoking prevention. Moreover, the delivery of the intervention within routine school settings enhances its potential applicability to other countries, particularly those with similarly high adult smoking prevalence or limited national resources. Finally, our study sample was well balanced between the intervention and control groups in terms of key sociodemographic factors and additional covariates, thereby minimizing the risk of confounding in the analysis.

This study has several limitations. First, the use of self-reported questionnaires may have introduced information bias, particularly for smoking behaviors and attitudes that are susceptible to social desirability. In addition, participants were recruited solely from the Thessaly region, which may limit the generalizability of the findings. Given the differential loss to follow-up between groups, a complete-case analysis may have introduced attrition bias. Although the observed smoking prevalence was lower than anticipated in the sample size calculation, raising concerns about potential underpowering, the study remained sufficiently powered to detect differences between the study groups. The latter suggests that the true strength of the association may be higher than observed in our study. The imbalance in participant numbers between the control and intervention arms reflects the cluster-randomized design, in which schools rather than individual students were randomized; such imbalances are common in school-based studies and do not inherently bias results when appropriately accounted for, as was done using GEE to adjust for clustering. Moreover, the interpretation of the results should be clearly confined to the one year follow up context and the age group of the participants. Finally, the study did not examine the use of novel tobacco products, such as e-cigarettes or heated tobacco devices, which should be addressed in future research.

## 5. Conclusions

In conclusion, the results of the present interventional study indicated significant improvements in the level of knowledge and attitudes towards smoking, as well as in the control of smoking initiation. Our findings support that integration of similar early prevention programs in school curricula could be beneficial for improving the above-mentioned outcomes and highlight the importance of continuous evaluation and adaptation for sustained impact. Integrating structured smoking prevention modules into elementary and early secondary school curricula could support long-term tobacco control efforts in countries with high smoking prevalence by enhancing positive anti-smoking behaviors from an early age.

## Figures and Tables

**Table 1:** Baseline characteristics of study participants.

Variable	Intervention Group	Control Group	ASD *
Gender			0.088
no. (%) male	161 (50.9%)	211 (46.6%)	
no. (%) female	155 (49.1%)	242 (53.4%)	
Age in years (SD)	11.24 (0.43)	11.29 (0.46)	0.11
Smoking of the mother, no. (%) yes	144 (36.9%)	147 (32.5%)	0.27
Smoking of the father, no. (%) yes	170 (43.5%)	171 (37.7%)	0.33
Smoking of any sibling, no. (%) yes	15 (3.8%)	17 (3.8%)	0.05
Smoking of any friend, no. (%) yes	10 (2.6%)	16 (3.5%)	0.02

* ASD: Absolute Standardized Difference; values of 0.2, 0.5, and 0.8 indicate small, medium, and large differences, respectively.

**Table 2:** Summary statistics of scores on the smoking knowledge–attitude scale across the three stages.

Stage	Mean ± SD	
	Intervention Group	Control Group
Baseline	60.36 ± 6.90	62.91 ± 5.90
2	62.88 ± 7.31	62.91 ± 6.85
3	62.27 ± 7.58	62.46 ± 6.45

**Table 3:** Mixed-effects multivariable linear regression results for baseline variables and the smoking knowledge–attitudes scale, including the time × group interaction.

Variable	F-Statistic	*p*-Value
Group	0.487	0.493
Stage	5.945	0.003
Stage × group	8.432	<0.001
Sex	8.828	0.003
Age	6.435	0.011
Smoking of the mother	3.613	0.057
Smoking of the father	0.084	0.772
Smoking of any sibling	0.033	0.856
Smoking of any friend	0.582	0.445

**Table 4:** Smoking prevalence (%) during baseline, 3 months post-intervention (stage 1) and 12 months post-intervention (stage 2).

Stage	Prevalence (%), *n*	
	Intervention Group	Control Group
Baseline	0.51 (*n* = 2)	0.22 (*n* = 1)
2	0.51 (*n* = 2)	0.44 (*n* = 2)
3	1.81 (*n* = 6)	2.68 (*n* = 12)

**Table 5:** Results of the multivariable GEE model examining the associations of baseline variables with smoking prevalence, as well as the effect of the stage × group interaction on changes in prevalence over time.

Variable	Wald Chi-Square	*p*-Value
Group	14.862	<0.001
Stage	9.347	0.002
Stage × group	6.425	0.011
Sex	0.001	0.992
Age	1.256	0.262

## Data Availability

The data presented in this study are available on request from the corresponding author.

## Supplementary Materials

The following supporting information can be downloaded at: https://www.mdpi.com/article/10.3390/ijerph23020270/s1. The English translation of the original questionnaire used in the study.

## Abbreviations

The following abbreviations are used in this manuscript:

COPD	Chronic obstructive pulmonary disease
GYTS	Global Youth Tobacco Survey
GEE	Generalized Estimating Equations
