# Supplementary material for: Evaluation of the Effectiveness of a School-Based Smoking Prevention Program Among Young Adolescents in Central Greece: An Analytical, Non-Randomized Interventional Study
Source: Int J Environ Res Public Health. 2026 Feb 22;23(2):270. doi: 10.3390/ijerph23020270 (PMC12941179; doi:10.3390/ijerph23020270)
Supplement: Supplementary file 1 [file ijerph-23-00270-s001.zip › ijerph-4069453-supplementary.pdf]

Questionnaire

Part A: Demographics

Day: Month: Year:

2. Are you a boy or a girl? ◻ Boy ◻ Girl

3. What is the name of your school?

4. What is the name of your class?

5. What is the name of the school you attended last school year?

6. Who lives together with you in your house?
(Tick all the answers that apply)

- My mother
- My father
- Other female carer (eg. Stepmotger, grandmother)
- Other male carer (eg. Stepfather, grandfather)
- One or more sisters (or stepsisters)
- One or more brothers (or stepbrothers)
- Other family members (eg. Uncle, grandparents)
- Other: (please specify)

7. Is your group of friends the same now as it was six months ago?
   - All my friends are the same as six months ago
   - Most of my friends are the same as six months ago
   - About half of my friends are the same as six months ago
   - More than half of my friends are the same as six months ago
   - All of my friends are new, compared to six months ago

8. How much money do you usually spend on your personal needs during a week?
   - I usually don’t have any money to spend.

- Less than 5 euros
- 5-9 euros
- 10-14 euros
- 15-19 euros
- 20-24 euros
- 25-29 euros
- More than 30 euros

		Yes	No	I do not know/don’t answer
9.	Does your mother smoke?			
10.	Does your father smoke?			
11.	Do any of your siblings smoke?			
12.	Do any of your friends smoke?			

Part B. Knowledges and attitudes related to smoking

What is your opinion about smoking?

1. Do you think that smoking..

- Relaxes someone very much
- Relaxes someone
- Relaxes someone a little
- It doesn’t help someone to relax
- I prefer not to answer

2. Do you think that smoking...

- is very harmful to health
- is harmful to health
- is probably harmful to my health
- is not harmful to my health
- I prefer not to answer

3. Smoking

- o It's silly
- o It's a bit silly
- o It's not silly at all
- o I don't know

4. Do you think that smoking...

- o relaxes someone a lot
- o relaxes someone
- o relaxes someone a little
- o neither relaxes nor stresses someone
- o stresses someone a little
- o stresses someone
- o stresses someone a lot
- o I don't know

5. Do you think that smoking

- o makes someone feel very confident in a group
- o makes someone feel confident in a group
- o makes someone feel somewhat confident in a group
- o neither gives nor takes away confidence
- makes someone feel less confident
- o I don't know

6. For someone to smoke is:

- o very wrong
- o wrong
- o a little wrong

- neither right nor wrong
- a little right
- right
- very right

7. Do teenagers who smoke have more friends?

- I completely agree
- I agree
- I don't know
- I disagree
- I completely disagree

8. Do you think that smoking means "independence"?

- I completely agree
- I agree
- I don't know
- I disagree
- I completely disagree

9. Does smoking make people your age "attractive"?

- I completely agree
- I agree
- I don't know
- I disagree
- I completely disagree

10. Does smoking help someone lose weight?

- I completely agree
- I agree
- I don't know

- I completely disagree
- I disagree

11. Is smoking a way to prove to others that you're "not afraid and are a risk-taker"?

- I completely agree
- I agree
- I don't know
- I completely disagree
- I disagree

12. Do people your age consider it normal for their peers to smoke?

- I completely agree
- I agree
- I don't know
- I disagree
- I completely disagree

13. Do you think that smoking can be "disgusting"?

- I completely agree
- I agree
- I don't know
- I completely disagree
- I disagree

14. Do you think that pregnant women who smoke can cause harm to the fetus?

- I completely agree

- I agree
- I don't know
- I completely disagree
- I disagree

15. Do young people who smoke run a greater risk of developing health problems in the future?

- I completely agree
- I agree
- I don't know
- I disagree
- I completely disagree

16. Do you believe that smoking is harmful to health?

- I completely agree
- I agree
- I don't know
- I completely disagree

I disagree

Part C. Use of smoking.

Have you smoked at all during the past month (even a single puff)?

Yes

No

I don't know/don't answer

Have you smoked at all during the past seven days (week) (even a single puff)?

Yes

No

I don't know/don't answer

Have you smoked at all during the past day (even a single puff)?

Yes

No

I don't know/don't answer
